# Glucagon-like peptide-1 receptor agonists are associated with fewer venous thromboembolic events and limb complications in obese patients with chronic venous insufficiency

**DOI:** 10.1016/j.jvsv.2026.102557

**Published:** 2026-06-20

**Authors:** Lavender Micalo, Mark Archie, Vincent L. Rowe, Adedunmola P. Adewale, Nathan Vu Pham, Albert Li, Juan Carlos Jimenez

**Affiliations:** a David Geffen School of Medicine, University of California, Los Angeles (UCLA), Los Angeles, CA; b Department of Surgery, Harbor-UCLA Medical Center, Torrance, CA; c Keck School of Medicine of the University of Southern California, Los Angeles, CA

**Keywords:** Agonist, Embolus, Glucagon-like peptide-1, Pulmonary, Receptor, Thrombosis, Venous

## Abstract

**Objective:**

Despite the well-documented impact of glucagon-like peptide-1 receptor agonist (GLP-1) therapy on obesity and diabetes, and the known risk factors for chronic venous insufficiency, the relationship between GLP-1 use and venous disease outcomes remains poorly understood. This study aims to assess the association between GLP-1 therapy and the risk of deep vein thrombosis (DVT), pulmonary embolism (PE), venous leg ulcers, cellulitis, and all-cause mortality at 1 year and 3 years following diagnosis in obese patients with chronic venous insufficiency (CVI).

**Methods:**

We conducted a retrospective multicenter cohort study using the TriNetX US Collaborative Network to identify adult patients (≥40 years) who were obese (body mass index ≥30 kg/m^2^) and had a diagnosis of CVI between 2016 and 2025. Patients who were started on GLP-1 therapy were matched 1:1 to a non–GLP-1 cohort using propensity scores that accounted for age, race, sex, smoking history, diabetes, body mass index, medications, and other comorbidities. Covariate balance was assessed using standardized mean differences, with values <0.1 indicating adequate balance. Patients with CVI and a prior history of DVT and PE were excluded from the study. The primary outcome was acute DVT risk at 1-year and 3-year follow-up after a CVI diagnosis. Secondary outcomes included PE, venous ulcers, associated soft-tissue infections, and all-cause mortality at both time points. Cox proportional hazard models were used to estimate hazard ratios (HRs) and 95% confidence intervals (CIs) for our outcomes. Time-to-event analyses were assessed using Kaplan–Meier survival curves, with intercohort comparisons performed using the log-rank test. Statistical significance was set at a two-sided α of .05. All analyses were conducted within the TriNetX Analytics platform.

**Results:**

Of the 138,853 obese patients with CVI included in our study, 12,874 patients (9.3%) were treated with GLP-1s. After propensity score matching, each cohort consisted of 12,379 patients with CVI and obesity. Following propensity score matching, GLP-1 therapy was associated with a decreased risk of acute DVT (HR, 0.54; 95% CI, 0.42-0.71; *P* < .001), PE (HR, 0.43; 95% CI, 0.27-0.66; *P* < .001), venous ulcers (HR, 0.47; 95% CI, 0.33-0.66; *P* < .001), and soft-tissue infections (HR, 0.61; 95% CI, 0.52-0.72; *P* < .001) at 1 year in patients with CVI and obesity. The association between GLP-1 therapy and reduced risk of developing DVT, PE, venous ulcers, and soft-tissue infections was sustained at the 3-year follow-up. At both 1- and 3-year follow-up, patients with treated with GLP-1 demonstrated a 4% (99.0% vs 94.0%; *P* < .001) and 9% (95.0% vs 86.0%; *P* < .001) absolute survival benefit, respectively, compared with their non–GLP-1 counterparts.

**Conclusions:**

In obese patients with CVI, GLP-1 therapy was associated with reduced risk of venous thromboembolic events, venous leg ulcers, and soft-tissue infections, as well as improved long-term survival. These findings suggest that GLP-1 use may confer clinically protective effects in patients with CVI. Future studies are warranted to better understand the role of GLP-1 treatment in the management of venous disease.

**Article Highlights**

• **Type of Research:** Retrospective review of prospectively collected TriNetX US Collaborative Network data
• **Key Findings:** In obese patients with chronic venous insufficiency, glucagon-like peptide-1 receptor agonist therapy was associated with reduced risk of venous thromboembolic events, venous ulcers, and soft-tissue infections, as well as improved long-term survival.
• **Take Home Message:** Glucagon-like peptide-1 receptor agonists may play a clinically beneficial role in reducing complications and improving survival in obese patients with chronic venous insufficiency.

Chronic venous insufficiency (CVI) is a worldwide public health problem, with studies estimating that 33% of the general population aged between 18 and 64 years of age are afflicted.^1^^,^^2^ In the United States alone, more than 25 million individuals have CVI, with more than six million with advanced stages of the disease.^3^ Although the relationship between CVI and the development of venous-related complications, including deep venous thrombosis (DVT), venous leg ulcers, and pulmonary embolus (PE) has not been definitively established, there is evidence that the presence of CVI may predispose to these serious sequelae contributing to increased morbidity, decreased quality of life, and increased mortality in a subset of these patients.^1^^,^^4^

Glucagon-like peptide-1 receptor agonists (GLP-1s) were initially developed to improve glycemic control in patients with type 2 diabetes mellitus and have also been shown to significantly promote weight loss.^5^ There is an increasing body of evidence demonstrating cardiovascular benefits of these medications, with multiple physiologic effects on blood vessels, including increased nitrous oxide production and vasodilation, decreased secretion of inflammatory cytokines, and reduced activity of matrix metalloproteinases.^6^^,^^7^ However, published evidence on the effects of GLP-1 in patients with CVI and its associated complications is sparse, and their true effects in these patients remain unknown.

Given the current knowledge gap in this area, our study aims to examine the relationship between GLP-1 and the risk of developing DVT, PE, venous leg ulcers, and soft-tissue infections, as well as all-cause mortality, in obese patients diagnosed with CVI. We hypothesized that GLP-1 therapy in patients with CVI who were obese is associated with a reduced hazard of venous thromboembolic events, wound complications, and all-cause mortality compared with patients who never received GLP-1 treatment.

## Methods

### Study design and data source

This retrospective cohort study used the TriNetX U.S. Collaborative Research Network (TriNetX LLC), a federated network of ∼68 health care organizations across the United States that provide access to deidentified electronic health record data. TriNetX also provides longitudinal clinical data on patient demographics, diagnoses, procedures, medications, and laboratory results. This data is mapped to the International Classification of Diseases, Tenth Revision (ICD-10), Current Procedural Terminology, Anatomical Therapeutic Chemical Classification System, Veterans Affairs National Formulary, RxNorm, and Logical Observation Identifiers Names and Codes. The study dataset was queried on January 1, 2026, and included encounters from January 1, 2016, to December 31, 2025. Because all data were deidentified, the study was deemed exempt from institutional review board review.

### Study population

Adult patients aged ≥40 years with a body mass index (BMI) ≥30 kg/m^2^ and diagnosed with CVI between 2016 and 2025 were identified using ICD-10 codes (Supplementary Table I, online only). These patients were then stratified into two groups, comprising those who had never been prescribed a GLP-1 (non–GLP-1 cohort) and those who had at least two GLP-1 prescriptions within 6 months of CVI diagnosis (GLP-1 cohort). RxNorm was used to identify GLP-1 prescriptions, including lixisenatide, albiglutide, dulaglutide, semaglutide, liraglutide, and exenatide. Patients with a history of DVT, PE, venous ulcers, or malignancy prior to a CVI diagnosis were excluded to minimize confounding from prevalent disease and ensure an incident complication cohort. Patients missing key demographic data, such as age and sex, were also excluded from the study.

### Covariates

Patient characteristics, including age, sex, race, and ethnicity, were defined using the TriNetX data dictionary. To identify pertinent comorbidities, established ICD-10 diagnosis codes were used. Comorbidities included hypertension, obesity, diabetes mellitus, ischemic heart disease, heart failure, peripheral arterial disease (PAD), chronic obstructive pulmonary disease, and tobacco use. Baseline BMI and hemoglobin A1c were also included to improve propensity score matching. Baseline medication use evaluated in our study included statins, diuretics, beta-blockers, angiotensin-converting enzyme inhibitors, anticoagulants, calcium channel blockers, angiotensin II receptor blockers, and other antihypertensive agents.

### Outcomes

All outcomes were identified using established ICD-10 diagnosis codes and assessed at 1 year and 3 years following a CVI diagnosis. The primary outcome was the acute risk of developing lower extremity DVT. Secondary outcomes included all-cause mortality risk and risk of developing PE, venous ulcers, and soft-tissue infections.

### Statistical analysis

Baseline demographic and clinical characteristics were summarized using descriptive statistics. Categorical variables were reported as counts and percentages and compared using Pearson χ^2^ tests, whereas continuous variables were reported as means ± standard deviations and compared using independent *t*-tests. To reduce confounding, 1:1 propensity score matching using greedy nearest-neighbor matching with a caliper of 0.1 pooled standard deviations was performed to create cohorts with matched covariates, as shown in Table. Covariate balance was assessed using standardized mean differences (SMDs), with values <0.1 indicating adequate balance.

**Table:** Baseline patient characteristics before and after propensity score matching (PSM)

Characteristic	Before matching				After matching		
	GLP-1 (n = 12,874)	Control (n = 125,979)	*P* value	SMD	GLP-1 (n = 12,379)	Control (n = 12,379)	SMD
Age, years	60.4 ± 10.3	64.7 ± 11.8	<.001	0.36	60.8 ± 10.3	61.2 ± 11.3	0.04
Sex							
Female	58.8	57.2	<.001	0.03	58.7	59.8	0.02
Male	41.2	42.8			41.3	40.2	
Race							
White	69.7	71.4	<.001	0.04	69.7	69.4	<0.01
Black	20.1	17.0	<.001	0.19	20.1	20.3	<0.01
Asian	1.4	1.5	.22	0.01	1.4	1.5	<0.01
Native Hawaiian	0.5	0.5	.87	<0.01	0.5	0.6	<0.01
American Indian	0.6	0.5	.61	<0.01	0.6	0.6	<0.001
Other race	3.1	3.9	<.001	0.04	3.1	3.2	0.01
Ethnicity							
Hispanic	8.7	11.3	<.001	0.09	8.7	8.6	<0.01
Non-Hispanic	71.5	68.7	<.001	0.06	71.4	72.4	0.02
Comorbidities							
HTN	82.8	58.5	<.001	0.55	82.3	84.1	0.06
Diabetes mellitus	68.8	27.7	<.001	0.92	67.6	67.9	0.02
IHD	27.1	20.3	<.001	0.27	27.5	29.2	0.03
Heart failure	19.0	14.5	<.001	0.26	24.3	20.0	0.03
Tobacco use	24.6	15.3	<.001	0.15	16.7	23.8	0.01
PAD	14.3	9.8	<.001	0.14	14.4	14.8	0.02
COPD	12.4	9.7	<.001	0.19	12.7	12.8	0.02
Baseline medications							
Statins	64.6	33.8	<.001	0.65	63.5	64.3	0.02
Diuretics	62.6	36.3	<.001	0.54	61.8	63.6	0.04
Beta blockers	49.4	32.6	<.001	0.35	49.3	50.8	0.03
ACE inhibitors	40.5	29.9	<.001	0.22	40.9	42.8	0.04
Anticoagulants	40.0	20.1	<.001	0.44	39.3	39.9	0.01
CCBs	38.7	23.8	<.001	0.33	38.6	39.2	0.01
ATII inhibitors	35.7	16.8	<.001	0.44	34.8	35.3	0.01
Antihypertensives	26.7	16.1	<.001	0.26	26.5	27.5	0.02
OCP	8.8	3.7	<.001	0.21	8.4	8.7	0.01
Hemoglobin A1c							
5.7-6.4 %	45.3	19.6	<.001	0.57	44.1	44.9	0.02
>6.5 %	54.6	13.6	<.001	0.96	52.9	51.8	0.02
BMI, kg/m^2^							
30-35	45.0	45.5	.30	<0.01	45.5	47.0	0.03
35-40	51.7	31.2	<.001	0.43	51.1	52.9	0.04
>40	60.0	25.5	<.001	0.74	58.5	59.1	0.01

*ATII*, Angiotensin II; *BMI*, body mass index; *CCB*, calcium channel blocker; *COPD*, chronic obstructive pulmonary disease; *GLP-1*, glucagon-like peptide-1 receptor agonist; *HTN*, hypertension; *IHD*, ischemic heart disease; *OCP*, oral contraceptives; *PAD*, peripheral arterial disease; *SMD*, standardized mean difference.

Data are presented as percent or mean ± standard deviation.

Outcomes were compared between matched cohorts without additional covariate adjustment, after achieving adequate balance across all covariates (all SMDs were <0.1). Effect estimates are reported as hazard ratios (HRs) with 95% confidence intervals (CIs). Time-to-event outcomes such as all-cause mortality were evaluated using Kaplan–Meier curves, with between-group comparisons performed using the log-rank test. Statistical significance was set at a two-sided α of .05. All analyses were conducted within the TriNetX Analytics platform.

## Results

**Fig 1:**
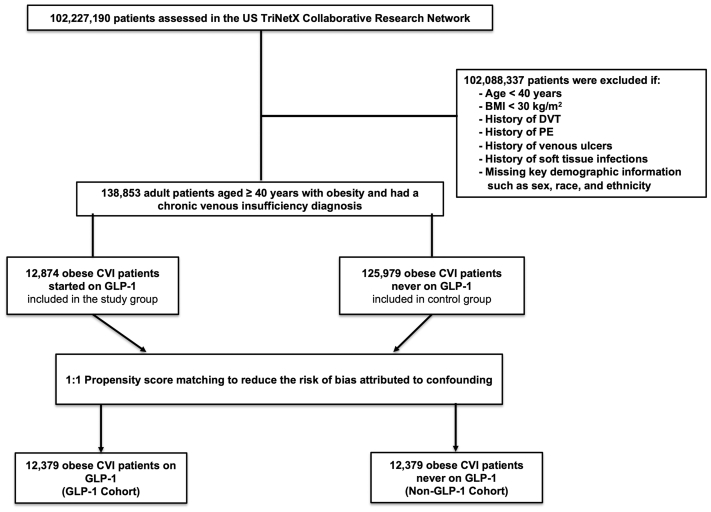
Study Consolidated Standards of Reporting Trials (CONSORT) diagram. *BMI*, Body mass index; *CVI*, chronic venous insufficiency; *DVT*, deep venous thrombosis; *GLP-1*, glucagon-like peptide-1 receptor agonist; *PE*, pulmonary embolism.

Of 138,853 obese patients with CVI, 12,874 patients (9.3%) were treated with a GLP-1 (Fig 1). Prior to matching, patients in the GLP-1 group were younger (60.4 ± 10.3 vs 64.7 ± 11.8 years; *P* < .001) but had higher rates of diabetes mellitus (68.8% vs 27.7%; *P* < .001), hypertension (82.8% vs 58.5%; *P* < .001), and PAD (14.3% vs 9.8%; *P* < .001) compared with their counterparts. Relative to the non–GLP-1 group, patients in the GLP-1 cohort had a higher prevalence of tobacco use (24.6% vs 15.3%; *P* < .001) and had a high proportion of patients with a BMI >40 kg/m^2^ (60.0% vs 25.5%; *P* < .001). Women represented nearly 58% of our study population (Table). After PSM, the control and experimental cohorts had 12,379 obese patients with CVI each (Fig 1). A balance of covariates was achieved across all variables. Summaries of baseline characteristics and SMDs for all covariates before and after matching are provided for each cohort in the Table.

**Fig 2:**
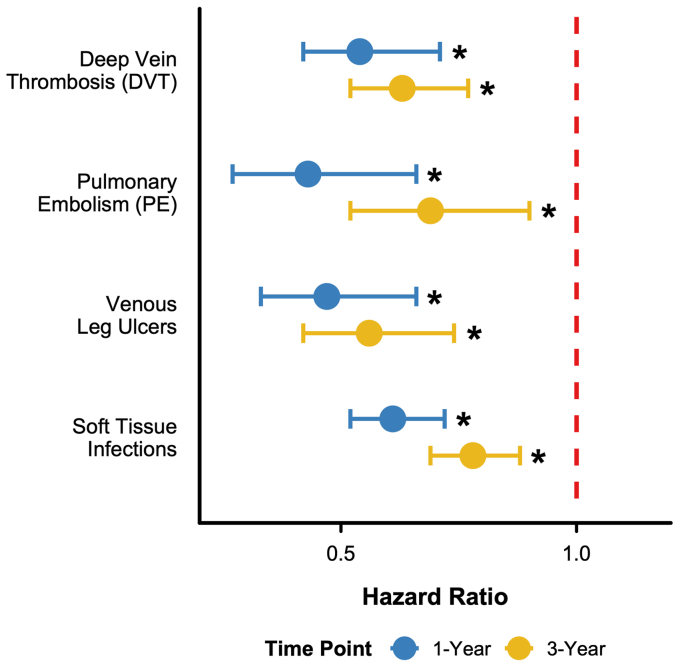
Cox regression analysis shows that glucagon-like peptide-1 receptor agonists (GLP-1s) are associated with a significant reduction in the risk of deep venous thrombosis (*DVT*), pulmonary embolism (*PE*), venous ulcers, and soft-tissue infections at 1 year and 3 years after a chronic venous insufficiency (CVI) diagnosis. ∗Indicates statistical significance (*P* < .05).

Post-matching, GLP-1 therapy was associated with a 46% (HR, 0.54; 95% CI, 0.42-0.71; *P* < .001) and 37% (HR, 0.63; 95% CI, 0.52-0.77; *P* < .001) reduced risk of developing DVT within 1 year and 3 years following a CVI diagnosis, respectively (Fig 2). Relative to the non–GLP-1 cohorts, patients with CVI who were obese and treated with GLP-1 had reduced hazards of PE (HR, 0.43; 95% CI, 0.27-0.66; *P* < .001), venous ulcers (HR, 0.47; 95% CI, 0.33-0.66; *P* < .001), and soft tissue infections (HR, 0.61; 95% CI, 0.52-0.72; *P* < .001) at 1-year follow-up.

Additionally, the GLP-1 group demonstrated a 31% lower PE hazard after 3 years relative to the non–GLP-1 group (HR, 0.69; 95% CI, 0.52-0.90; *P* < .01). Furthermore, patients in the GLP-1 cohort had a 44% (HR, 0.56; 95% CI, 0.42-0.74; *P* < .001) and a 22% (HR, 0.78; 95% CI, 0.69-0.88; *P* < .001) reduced risk of developing venous ulcers and soft-tissue infections within 3 years of a CVI diagnosis, respectively.

**Fig 3:**
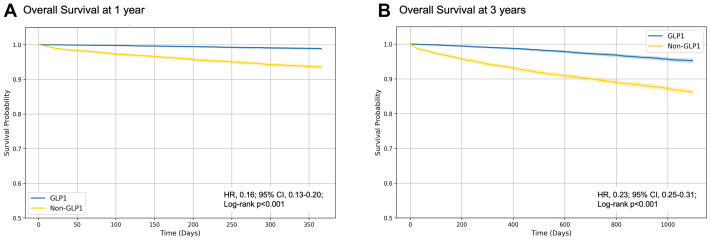
Kaplan–Meier time-to-event analysis demonstrating overall survival at 1 and 3 years among patients with obesity and chronic venous insufficiency (CVI). Shaded areas indicate 95% confidence intervals (*CIs*). Patients treated with glucagon-like peptide-1 receptor agonists (*GLP-1s*) demonstrated significantly improved overall survival at 1 and 3 years compared with those not treated with GLP-1 (log rank *P* < .001). At 1-year follow-up **(A)**, overall survival rates in the GLP-1 and non–GLP-1 cohorts were 99% and 94%, respectively. At 3-year follow up **(B)**, overall survival rates in the GLP-1 and non–GLP-1 cohorts were 95% and 86%, respectively. *HR*, Hazard ratio.

Notably, GLP-1 treatment was also associated with a reduced hazard of mortality (HR, 0.16; 95% CI, 0.13-0.20; *P* < .001) and a higher survival rate (99.0 vs 94.0%; χ^2^ = 429.4) (Fig 3, *A*) at 1-year follow-up. This mortality benefit associated with GLP-1 therapy was sustained after 3 years of CVI diagnosis (HR, 0.23; 95% CI, 0.25-0.31; *P* < .001) (Fig 3, *B*), with a 9% absolute survival benefit (95.0% vs 86.0%; χ^2^ = 488.2) compared with their counterparts. Supplementary Table I (online only) lists all ICD-10 codes used in our data analysis.

## Discussion

Despite the current widespread use of GLP-1 therapy, its role in CVI outcomes remains minimally explored. This is the first study to report these outcomes in a cohort comprised entirely of obese patients with CVI. Using robust PSM, we evaluated the impact of GLP-1 on venous disease outcomes and overall survival among obese patients diagnosed with CVI. GLP-1 therapy was associated with a reduced risk of developing acute DVT, PE, venous ulceration, and soft-tissue infections, with benefits sustained for 3 years. Patients with CVI treated with GLP-1s demonstrated greater survival at 1 and 3 years relative to their untreated counterparts. With implications for clinical practice, several of these findings merit further discussion.

Over the study period, obese patients with CVI receiving GLP-1 therapy had a 46% and 37% reduced risk of acute DVT at 1-year and 3-year follow-up, respectively. Existing literature on GLP-1s and their impact on DVT present conflicting results that warrant careful interpretation. For instance, a 2025 target trial emulation study involving 540,258 patients with type 2 diabetes found that GLP-1 use was associated with a 19% lower risk of DVT compared with DPP-4 inhibitors.^8^ Conversely, a recent meta-analysis of 39 randomized controlled trials involving 70,499 patients found that GLP-1s were associated with a 64% increased risk of DVT, with nearly twice the DVT risk noted after 1.5 years of treatment.^9^ However, it is important to note that this meta-analysis primarily enrolled patients with a more diverse set of comorbidities, including diabetes, liver disease, and metabolic syndrome, compared with our CVI-specific study population. The reduced DVT risk observed in our study may reflect the higher baseline thrombotic risk in patients with CVI, due to chronic venous stasis and endothelial dysfunction, thereby increasing the likelihood of detecting protective effects. Furthermore, the pathophysiology of DVT in CVI, driven by venous stasis and local inflammation, differs from that reported in arterial-predominant populations, suggesting GLP-1 therapy may confer distinct benefits in venous-specific disease. Hence, our findings suggest that GLP-1 effects may vary markedly by clinical context, underscoring the need for population-specific studies, particularly in CVI cohorts where venous stasis and obesity-driven pathophysiology predominate.

Obesity is an established risk factor for thrombotic complications through venous stasis, hypercoagulability, and transmission of intra-abdominal pressure to leg veins, leading to endothelial damage and valvular dysfunction.^10^, ^11^, ^12^, ^13^ A prospective study by Willenberg and colleagues demonstrated that obese individuals had a 19.7% increase in femoral vein diameter, which was associated with reduced venous flow velocities and shear stress: major risk factors of venous thromboembolic events.^14^ Moreover, abdominal obesity has been linked to increased intra-abdominal pressure transmitted to the leg veins, causing greater tension on the venous wall and, consequently, reducing venous elasticity.^14^ Taken together, our findings highlight the potential role of GLP-1s in the management of CVI given their well-established impact on weight loss.

Beyond weight reduction, multiple molecular-level physiological factors may contribute to the clinical benefits demonstrated in our analysis. The pathogenesis of CVI is thought to be primarily driven by venous hypertension and chronic inflammation, marked by leukocyte infiltration of the venous wall and valve leaflets, triggering the release of proinflammatory cytokines and activation of matrix metalloproteinases, leading to extracellular matrix degradation, venous wall remodeling, and valve incompetence.^8^^,^^15^, ^16^, ^17^ In the present study, we noted that obese patients with CVI receiving GLP-1 treatment had a lower hazard of developing venous ulcers and soft-tissue infections at both time points. These findings are congruent with current literature reporting the physiological effects of GLP-1 therapy on vascular endothelium, which may help explain some of our observations.^16^^,^^17^ Narvaez et al found that diabetic patients treated with GLP-1s had a shorter median healing time for venous ulcers at 90 days, although this benefit was not sustained at long-term follow-up.^18^ Similarly, Go and colleagues noted that among patients with an active venous ulcer, GLP-1 use was associated with a nearly 20% reduction in the risk of nonhealing ulcers and soft-tissue infections at 1-year follow-up.^19^ Some have suggested that these medications promote wound healing by upregulating vascular endothelial growth factor expression, enhancing angiogenesis, and improving blood supply to ischemic tissues, all of which are crucial to wound healing.^20^, ^21^, ^22^ GLP-1s have also been implicated in the upregulation of endothelial nitric oxide synthase expression, leading to increased nitric oxide production and endothelium-dependent vasodilation.^22^ Furthermore, these medications also decrease reactive oxygen radical production and reduce endothelial damage.^23^ Risk factors such as chronic inflammation are central to CVI pathophysiology and contribute to venous wall remodeling, valve dysfunction, and ulcer development.^24^ Of note, anti-inflammatory effects at the level of the vessel wall also occur with GLP-1 use, lowering the vascular expression of inflammatory mediators and decreasing leukocyte infiltration.^24^ Thus, these findings further support our conclusion that GLP-1s confer protective benefits to patients with CVI beyond weight management and glycemic control.

Consistent with prior literature, GLP-1 use was associated with 84% and 77% reductions in the hazard of all-cause mortality at 1-year and 3-year follow-up, respectively.^25^ Moreover, obese patients with CVI starting GLP-1 therapy in our study experienced 4% and 9% absolute survival benefits at 1-year and 3-year follow-up, respectively, compared with their counterparts. A meta-analysis of 21 randomized controlled trials investigating the efficacy and safety of GLP-1s in high-risk patients with comorbid diabetes, kidney failure, heart failure, and high BMI reported a 12% reduction in all-cause mortality among patients treated with GLP-1 compared with placebo.^26^ In patients with PAD, GLP-1s have been associated with a 45% lower all-cause mortality and, more specifically, a 29% lower mortality risk compared with sodium-glucose cotransporter 2 inhibitors.^27^ The large effect sizes observed in our study may partially reflect that patients prescribed GLP-1s often have better access to health care or possess unmeasured characteristics associated with improved survival.^28^ Additionally, the higher prevalence of diabetes and hypertension in our GLP-1 cohort further supports the interpretation that these patients may already be engaged with the health care system for chronic disease management, potentially conferring surveillance benefits independent of medication effects. Lastly, our CVI-specific population may also derive particular benefit from GLP-1 therapy, given the central role of obesity and inflammation in CVI pathophysiology, with potential synergistic benefits including weight loss, enhanced endothelial function, and anti-inflammatory effects.^26^^,^^29^ Nonetheless, the directional consistency between randomized and observational studies, and across general and vascular-specific populations, strengthens our conclusion that GLP-1s are associated with meaningful reductions in all-cause mortality. It should also be noted that the included studies report different effect size measures, which are related but not identical estimates, and direct numerical comparisons across studies should be interpreted with this limitation in mind. Prospective studies specifically designed for CVI populations are needed to confirm these associations.

This study has several limitations inherent to its retrospective design and use of TriNetX, an administrative database. The TriNetX database is susceptible to different coding practices across participating health care organizations, which may introduce coding inaccuracies or misclassification bias. To minimize these confounding variables, we employed robust statistical methods, such as propensity matching. Furthermore, the study’s observational nature limits causal inference and may include residual confounding. Additionally, TriNetX lacks granular data on GLP-1 dosage, medication adherence, and lifestyle interventions. BMI can be an inaccurate measure of overweight status because it does not distinguish between fat and lean mass. We could not assess clinical severity, Clinical Etiological Anatomical Pathophysiology (CEAP) classification, or anatomic distribution of venous disease; factors that influence both treatment decisions and outcomes. Additionally, the ICD-10 code for venous insufficiency is not specific to these variables. Notably, our study cannot fully distinguish the effects of GLP-1 therapy from concomitant decreased morbidity linked to the weight loss these medications promote. However, emerging evidence from studies such as the SELECT trial suggests that GLP-1 treatment may confer clinical benefits independent of weight loss.^30^ It is also important to note that for the GLP-1 cohort, the index date was defined as the later of either the CVI diagnosis or the initiation of GLP-1 prescription in the main analysis. Although this approach ensured that only 1-year and 3-year outcomes occurring after initial medication exposure were captured, it may have introduced potential immortal-time bias that could overestimate these protective associations. However, the consistency of findings across multiple outcomes and their biological plausibility of associations support a true protective effect. Lastly, generalizability may be limited to patients within the included health care systems and may not extend to all patients with CVI.

## Conclusions

Our study, using data from a multicenter, nationally representative database, found that GLP-1 use among obese patients with CVI was linked to a significant decrease in the risk of acute DVT, PE, venous ulcers, and soft-tissue infections over the study period. GLP-1 therapy was also associated with improved survival at 1- and 3-year follow-up. Given the limited granularity inherent to TriNetX, these promising outcomes should be interpreted as hypothesis-generating. Future studies are warranted to validate these associations and to increase understanding of the potential role of GLP-1s in managing patients with CVI.

## Author Contributions

Conception and design: LM, MA, VR, JJ

Analysis and interpretation: LM, MA, VR, AA, NP, AL, JJ

Data collection: LM. AA, NP, AL

Writing the article: LM, MA, VR, AA, NP, AL, JJ

Critical revision of the article: LM, MA, VR, AA, NP, AL, JJ

Final approval of the article: LM, MA, VR, AA, NP, AL, JJ

Statistical analysis: LM, AA, NP, AL

Obtained funding: Not applicable

Overall responsibility: JJ

## Funding

None.

## Disclosures

J.C.J. is an advisory board member for Boston Scientific (100% of earnings donated to charitable foundations). The remaining authors report no conflicts.
