# Supplementary material for: Glucagon-like peptide-1 receptor agonists are associated with fewer venous thromboembolic events and limb complications in obese patients with chronic venous insufficiency
Source: J Vasc Surg Venous Lymphat Disord. 2026 Jun 20;14(5):102557. doi: 10.1016/j.jvsv.2026.102557 (PMC13383999; doi:10.1016/j.jvsv.2026.102557)
Supplement: Supplementary Table I [file mmc1.docx]

**SUPPLEMENTAL TABLE 1: ICD-10 CODES USED IN GLP-1RA AND CHRONIC VENOUS INSUFFICIENCY STUDY**

**INCLUSION CRITERIA**

| **Category** | **ICD-10 Code** | **Description** |
| --- | --- | --- |
| Primary Diagnosis | I87.2 | Venous insufficiency (chronic) (peripheral) |

| **Category** | **RxNorm** |
| --- | --- |
| Lixisenatide | 1440051 |
| Semaglutide | 1991302 |
| Tirzepatide | 2601723 |
| Liraglutide | 475968 |
| Exenatide | 60548 |
| Dulaglutide | 1551291 |

**EXCLUSION CRITERIA**

***Malignancy***

| **Category** | **ICD-10 Code** | **Description** |
| --- | --- | --- |
| Neoplasms | C00-D49 | All neoplasms |

***Venous Ulcers***

| **Category** | **ICD-10 Code** | **Description** |
| --- | --- | --- |
| Chronic Venous Hypertension | I87.31 | Chronic venous hypertension (idiopathic) with ulcer |
| Post-thrombotic Syndrome | I87.01 | Postthrombotic syndrome with ulcer |
| Chronic Venous Hypertension | I87.33 | Chronic venous hypertension (idiopathic) with ulcer and inflammation |
| Post-thrombotic Syndrome | I87.03 | Postthrombotic syndrome with ulcer and inflammation |

***Soft Tissue Infections***

| **Category** | **ICD-10 Code** | **Description** |
| --- | --- | --- |
| Cellulitis/Abscess - ICD-9 | 682.6 | Cellulitis and abscess of leg, except foot |
| Cellulitis/Abscess - ICD-9 | 682.7 | Cellulitis and abscess of foot, except toes |
| Cellulitis - Right Lower Limb | L03.115 | Cellulitis of right lower limb |
| Cellulitis - Left Lower Limb | L03.116 | Cellulitis of left lower limb |
| Acute Lymphangitis - Right | L03.125 | Acute lymphangitis of right lower limb |
| Acute Lymphangitis - Left | L03.126 | Acute lymphangitis of left lower limb |
| Post-thrombotic with Inflammation | I87.02 | Postthrombotic syndrome with inflammation |
| Chronic Venous Hypertension | I87.32 | Chronic venous hypertension (idiopathic) with inflammation |
| Post-thrombotic Ulcer/Inflammation | I87.03 | Postthrombotic syndrome with ulcer and inflammation |
| CVH Ulcer/Inflammation | I87.33 | Chronic venous hypertension with ulcer and inflammation |

***Deep Vein Thrombosis***

| **Category** | **ICD-10 Code** | **Description** |
| --- | --- | --- |
| DVT - Lower Extremity | I82.4 | Acute embolism and thrombosis of deep veins of lower extremity |
| Superficial Vein Thrombosis | I82.81 | Embolism and thrombosis of superficial veins of lower extremities |
| Other Venous Thrombosis | I82.89 | Embolism and thrombosis of other specified veins |
| Inferior Vena Cava Thrombosis | I82.220 | Acute embolism and thrombosis of inferior vena cava |
| Phlebitis - Unspecified Site | I80.9 | Phlebitis and thrombophlebitis of unspecified site |
| Phlebitis - Deep Vessels LE | I80.2 | Phlebitis and thrombophlebitis of other and unspecified deep vessels of lower extremities |
| Phlebitis - Femoral Vein | I80.1 | Phlebitis and thrombophlebitis of femoral vein |
| Phlebitis - LE Unspecified | I80.3 | Phlebitis and thrombophlebitis of lower extremities, unspecified |

***Pulmonary Embolism***

| **Category** | **ICD-10 Code** | **Description** |
| --- | --- | --- |
| PE with Cor Pulmonale | I26.0 | Pulmonary embolism with acute cor pulmonale |
| PE without Cor Pulmonale | I26.9 | Pulmonary embolism without acute cor pulmonale |
| Air Embolism | T80.0XXA | Air embolism following infusion, transfusion, and therapeutic injection, initial encounter |
| Venous Complication Post-Procedure | T81.72XA | Complication of vein following a procedure, not elsewhere classified, initial encounter |
| Arterial Complication Post-Procedure | T81.718A | Complication of other artery following a procedure, not elsewhere classified, initial encounter |
| Cardiac Device Embolism | T82.817A | Embolism due to cardiac prosthetic devices, implants and grafts, initial encounter |
| Vascular Device Embolism | T82.818A | Embolism due to vascular prosthetic devices, implants and grafts, initial encounter |
